# Qualitatively and Quantitatively Different Configurations of Nematic–Nanoparticle Mixtures

**DOI:** 10.3390/nano14050436

**Published:** 2024-02-27

**Authors:** Maha Zid, Kaushik Pal, Saša Harkai, Andreja Abina, Samo Kralj, Aleksander Zidanšek

**Affiliations:** 1 Jožef Stefan Institute, Jamova cesta 39, 1000 Ljubljana, Sloveniasasa.harkai@ijs.si (S.H.);; 2 Jožef Stefan International Postgraduate School, Jamova cesta 39, 1000 Ljubljana, Slovenia; 3 University Centre for Research and Development (UCRD), Department of Physics, Chandigarh University, Ghruan, Mohali 140413, Punjab, India; 4 Faculty of Mathematics and Physics, University of Ljubljana, Jadranska cesta 19, 1000 Ljubljana, Slovenia; 5 Faculty of Natural Sciences and Mathematics, University of Maribor, Koroška cesta 160, 2000 Maribor, Slovenia

**Keywords:** nanoparticles, nematic liquid crystals, point and line defects, topological charge

## Abstract

We consider the influence of different nanoparticles or micrometre-scale colloidal objects, which we commonly refer to as particles, on liquid crystalline (LC) orientational order in essentially spatially homogeneous particle–LC mixtures. We first illustrate the effects of coupling a single particle with the surrounding nematic molecular field. A particle could either act as a “dilution”, i.e., weakly distorting local effective orientational field, or as a source of strong distortions. In the strong anchoring limit, particles could effectively act as topological point defects, whose topological charge *q* depends on particle topology. The most common particles exhibit spherical topology and consequently act as *q* = 1 monopoles. Depending on the particle’s geometry, these effective monopoles could locally induce either point-like or line-like defects in the surrounding LC host so that the total topological charge of the system equals zero. The resulting system’s configuration is topologically equivalent to a crystal-like array of monopole defects with alternating topological charges. Such configurations could be trapped in metastable or stable configurations, where the history of the sample determines a configuration selection.

## 1. Introduction

Mixtures of diverse nanoparticles (NPs) and LCs [1,2,3] are typical representatives of complex systems [1,2,3,4,5,6], offering a rich pallet of qualitatively different configurations. These could enable numerous applications, particularly in photonics, sensitive detectors, and memory devices, to mention some of them [5,7]. Furthermore, such systems often exhibit diverse universal features [8,9,10,11,12,13] and could be exploited as relatively easy and cheap experimentally accessible testing grounds to study different open problems in physics.

LCs are unique materials that combine the properties of ordinary liquids and crystals. They consist of weakly interacting anisotropic molecules, which can display macroscopic orientational and, in some cases, translational order, while mass centres exhibit liquid-like behaviour. For illustration purposes, we limit our discussion to uniaxial rod-like LC molecules, which can exhibit uniaxial long-range orientational order in bulk equilibrium. The resulting nematic liquid crystal (NLC) phase represents the simplest LC configuration [1,2]. At large enough temperatures, it enters isotropic (I), the ordinary liquid phase. Upon decreasing the temperature, NLCs exhibit a weak first-order phase transition into the nematic phase. The resulting order is at the lowest approximation modelled by the uniaxial nematic order parameter *S* and the nematic director field $\hat{n}$. Here, *S* determines the degree of nematic uniaxial order, where *S* = 0 in the isotropic phase. 

Furthermore, the unit pseudovector $\hat{n}$ points along the local mesoscopic uniaxial direction, where the states $\pm \hat{n}$ [1,2] are physically equivalent (the so-called head-to-tail symmetry). In bulk equilibrium, both nematic fields are spatially homogeneous and $\hat{n}$ is aligned along a symmetry-breaking direction. 

At the I–N phase transition, the continuous symmetry of the high-temperature isotropic phase is broken. Consequently, low-temperature nematic phases possess Goldstone modes [1,3] and LC order is extremely susceptible to diverse perturbations. Therefore, one can efficiently manipulate nematic order by immersing different NPs in the LC fluid host. The resulting emergent system can generally exhibit new effective materials, yielding qualitatively different material or functional properties compared to those of pure NLCs. Due to liquid LC behaviour, one can relatively efficiently immerse different NPs into the LC host [14,15]. 

Furthermore, by combining appropriate LCs and NPs, one could sensitively tune the desired properties of the resulting effective system. Therefore, one can combine unique LC properties (softness, liquid character, orientational order, and optical transparency) [3,16] with additional desired properties which are introduced by NPs [14,17,18]. The latter can consist of diverse materials (conductors, isolators, semiconductors, etc.) and display different geometries. For example, they could be point-, line-, or platelet-like, possessing different symmetries.

In addition, in the strong anchoring regime, particles could effectively act as topological point defects [19]. Suppose the total topological charge of the system equals zero, which is, in typical cases, energetically favourable. In this case, such particles will introduce numerous topological defects (TDs) in the system in the NLC host to compensate for the particles’ enforced topological charge. The resulting TDs could emerge as either point or line defects. The latter could form diverse complex configurations, including knot-like structures [8,20,21,22,23]. 

Consequently, a rich diversity of qualitatively and qualitatively different structures could emerge [24,25,26]. Often, the resulting effective systems exhibit glass-like particle-tunable features. For example, in aerosil–NLC mixtures, one could obtain at least three qualitatively different disorder regimes just by varying the concentrations of aerosils [27,28,29,30]. The anchoring strength determines the interaction between NPs and LCs at low concentrations of NPs, and at higher concentrations of NPs, the NPs get closer to one another; thus, the ideal structures are modified and differ from those at low concentrations of NPs.

Furthermore, different structures could be stabilised by varying the conditions (i.e., history) of an NLC–particle mixture preparation [31,32]. On the contrary, if strong LC-mediated directional coupling occurs between LC-immersed anisotropic particles, crystal-like structures of particles exhibiting different symmetries could be established [9,33,34]. Recent developments in particle synthesis allow for the formation of almost arbitrary shapes of colloidal objects and nanoparticles. Consequently, by choosing appropriate NLC–particle mixtures with a well-defined particle geometry, one could predetermine and manipulate the desired symmetry of the effective system. For example, it is enormously challenging to create biaxial NLCs [9] in simple mono-molecular LCs. Such systems could lead to the development of fast-switchable systems between optically different states using relatively minute external electric fields, which is interesting for developing displays. Recent studies [9] revealed that such systems could be established in homogeneous mixtures of rod-like LC molecules and cylindrically shaped micrometre-sized colloids. There is a strong interest in establishing an appropriate combination of NLCs and appropriately shaped and surface-decorated particles of a proper size, which could be assembled into predetermined crystal-like structures mimicking classical atomic-base crystals. Furthermore, even particle-formed crystals exhibiting symmetries not yet encountered in nature could be formed by choosing appropriately shaped particles.

In the present manuscript, we focus on the structural and phase behaviour of NLC and NP mixtures, where NPs are more or less homogeneously dispersed in the LC host.

## 2. Mesoscopic Modelling

We first introduce a simple Landau-type mesoscopic model, which we use to interpret selected physical phenomena encountered in NLC–NP mixtures. We describe local nematic order using symmetric and traceless tensor order parameters [1]

(1) $$\underline{Q}=\sum_{i=1}^{3}s_{i}{\hat{e}}_{i}⊗{\hat{e}}_{i}.$$

Here, $s_{i}$ and ${\hat{e}}_{i}$ stand for the *i*th $\underline{Q}$ eigenvalue and eigenvector, respectively. In the case of uniaxial states, it can be expressed as [1]

(2) $$\underline{Q}=S\left(\hat{n}⊗\hat{n}-\underline{I}/3\right),$$

where *S* and $\hat{n}$ stand for the uniaxial nematic order parameter and the nematic director field, respectively.

We consider cases where NPs are diluted in an NLC medium. Their volume concentration is given by [1,2,35]

(3) $$\phi =\frac{N_{NP}v_{NP}}{V}$$

where $N_{NP}$ stands for the number of NPs in volume *V* and $v_{NP}$ is the volume of an average NP.

In terms of $\underline{Q}$, we express the free energy of the system as

(4) $$F=\int f_{V}d^{3}\vec{r}+\sum_{j}\int f_{i}^{\left(j\right)}d^{2}\vec{r}$$

Here, the volume LC free energy density $f_{V}=f_{c}{+f}_{e}+f_{f}$ consists of condensation ($f_{c}$), elastic ($f_{e}$) and an external electric field ($f_{f}$) contribution. Furthermore, the interfacial free energy density $f_{i}^{\left(j\right)}$ describes the local short-range interaction between the *j*th NP and nearby LC molecule. Henceforth, we assume that all NPs are identical. The free energy densities are given by [1,2,36]

(5) $$f_{c}=\frac{3a_{0}(T-T^{*})}{2}Tr{\underline{Q}}^{2}-\frac{9b}{2}Tr{\underline{Q}}^{3}+\frac{9c}{4}{\left(Tr{\underline{Q}}^{2}\right)}^{2}$$

(6) $$f_{e}=L\;Tr{\left(\nabla \underline{Q}\right)}^{2}$$

(7) $$f_{f}=-\frac{3}{2}\varepsilon_{0}\Delta \varepsilon \vec{E}.\underline{Q}\vec{E},$$

(8) $$f_{i}^{\left(j\right)}=w_{1}^{\left(1\right)}\;{\hat{e}}_{i}.\underline{Q}{\hat{e}}_{i}+w_{1}^{\left(2\right)}\;Tr{\underline{Q}}^{2}+w_{2}^{\left(2\right)}\;{\left({\hat{e}}_{i}.\underline{Q}{\hat{e}}_{i}\right)}^{2}+w_{3}^{\left(2\right)}\;\underline{Q}{\hat{e}}_{i}.\underline{Q}{\hat{e}}_{i}\;.$$

The numerical coefficients in Equations (5)–(8) are introduced for later convenience. We use a minimal model, considering only the most essential terms needed to illustrate the phenomena of our interest. Quantities $a_{0}$, *b*, and *c* are Landau expansion material dependent coefficients, $T^{*}$ stands for the bulk isotropic phase supercooling temperature, *L* is the representative bare (i.e., temperature independent) LC elastic constant (we consider the single elastic constant approximation), $\vec{E}=E{\hat{e}}_{f}$ is an external electric field aligned along the unit vector ${\hat{e}}_{f}$, Δε stands for the dielectric anisotropy (we limit to material with positive anisotropy), and $\varepsilon_{0}$ is the vacuum electric permittivity. The term $f_{i}^{\left(j\right)}$ describes the most general form of the interfacial free energy contribution of the *j*th NP up to the fourth order in $\underline{Q}$, where one assumes that a local interface imposes a single characteristic symmetry breaking direction ${\hat{e}}_{i}.$ Quantities $w_{1}^{\left(1\right)}$, $w_{1}^{\left(2\right)},$ $w_{2}^{\left(2\right)},$ and $w_{3}^{\left(2\right)}$ describe material-dependent properties.

We briefly comment on the physical meaning of the free energy terms. The condensation term enforces a 1st order phase transition at the critical temperature $T_{IN}=T^{*}+\frac{b^{2}}{4a_{0}c}.$ The equilibrium order parameter is given by $S_{eq}(T\leq T_{IN})=S_{0}\left(3+\sqrt{9-8(T-T^{*})/(T_{IN}-T^{*})}\right)$ and $S_{eq}\left(T>T_{IN}\right)=0,$ where $S_{0}=\frac{b}{2c}.$ The elastic term enforces spatially homogeneous nematic order. The external field term tends to align with the nematic order along $\vec{E}$.

The surface free energy contributions described by Equation (8) mimic diverse LC–substrate interactions. The most common surface-imposed terms reported are expressed as [36,37,38,39]

(9) $$f_{i}^{\left(j\right)}=-\frac{3}{2}w{\hat{e}}_{i}.\underline{Q}{\hat{e}}_{i}$$

or

(10) $$f_{i}^{\left(j\right)}=\frac{3}{2}wTr{\left(\underline{Q}-{\underline{Q}}^{\left(i\right)}\right)}^{2}.$$

The form given in Equation (9) has an external field-like structure (see Equation (7)). For w>0, the NP–LC interface enforces local order along $\pm {\hat{e}}_{i}$ and *S* > 0. On the contrary, for w<0, orientations perpendicular to ${\hat{e}}_{i}$ are favoured and *S* < 0. In Equation (10), one assumes w>0 and ${\underline{Q}}^{\left(i\right)}$ describes the NP–LC interface favoured order. From Equation (8), one reproduces Equation (9) for $w_{1}^{\left(1\right)}=-\frac{3}{2}w$ and $w_{1}^{\left(2\right)}=w_{2}^{\left(2\right)}=w_{3}^{\left(2\right)}=0.$ Furthermore, one obtains Equation (10) for the following set of parameters:

$$w_{1}^{\left(1\right)}=-\frac{3}{2}w\;,\;w_{7}^{\left(2\right)}=S_{i}w/3,\;w_{2}^{\left(2\right)}=w_{2}^{\left(3\right)}=0\;,\;\mathrm{where}\;S_{i}=\sqrt{{\frac{3}{2}Tr{\underline{Q}}^{\left(i\right)}}^{2}}.$$

Note that different values of surface interaction constants reflect different material and chemical properties describing LC–NP coupling.

## 3. Phase and Structural Behaviour 

**Figure 1:**
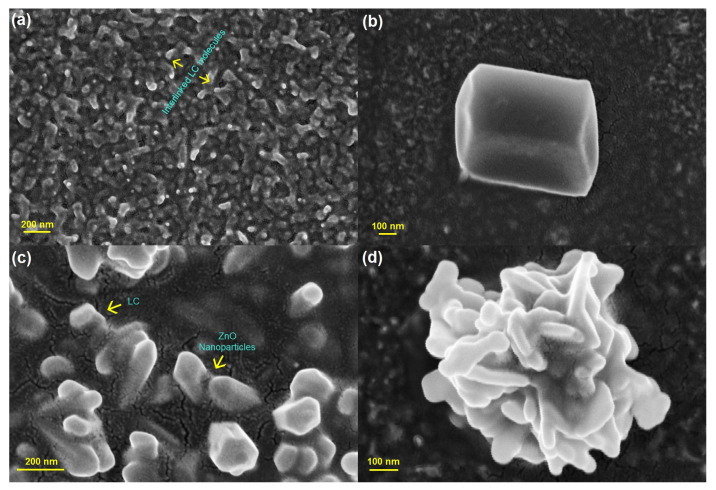
Structural investigation of the ZnO nanoparticles dispersed into liquid crystalline materials through typical Field Emission Scanning Electron Microscopy (FE-SEM) images. (**a**) Warm-like structures evolved in the system of interlinked liquid crystalline molecules. (**b**) A typical hexagonal ZnO nanoparticle. (**c**) ZnO NPs dispersed into liquid crystals hybrid composite matrix. (**d**) Liquid crystalline NP embedded system can lead to flower-like structures.

We first focus on the interaction of individual NPs with the LC order. For example, ZnO nanoparticles dispersed in liquid crystalline optical materials can be observed through Field Emission Scanning Electron Microscopy (FE-SEM). These FE-SEM structural investigations can depict hexagonal interlinked processes. Here, Figure 1a presents interlinked liquid crystalline molecules, where warm-like structures evolved. A typical hexagonal ZnO nanoparticle is shown in Figure 1b. The case of ZnO NPs dispersed into liquid crystals hybrid composite matrix is shown in Figure 1c. Flower-like structures of the liquid crystalline NP embedded system at the highest magnification are presented in Figure 1d.

The key measure of NP–LC coupling is the ratio [23,24]

(11) $$\mu =\frac{RW}{K}\;,$$

where *R* stands for the characteristic linear size of a NP, W stands for the characteristic anchoring strength at the NP–LC interface, and $K\sim LS^{2}$ is the representative Frank elastic constant. The condition μ~1 roughly separates regimes where NPs weakly (μ≪1) and strongly (μ≫1) perturb the surrounding nematic director field. 

**Figure 2:**
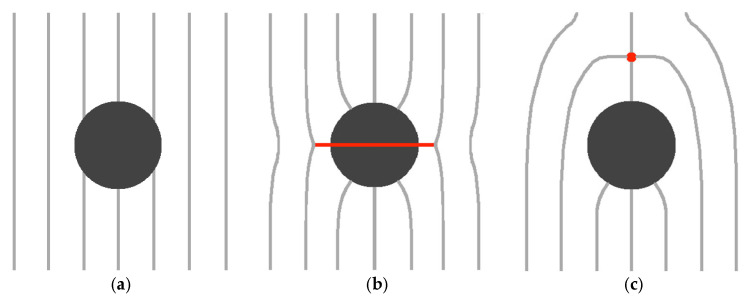
Schematic presentation of different nematic structures imposed by a spherical particle, which enforces a homeotropic anchoring condition. In the case of strong anchoring, the particle acts as a point defect bearing topological charge *q* = 1. (**a**) Extremely weak anchoring condition. In this case, the NP does not disturb the nematic director field and is essentially spatially homogeneous. (**b**) Strong anchoring condition. A possible solution is a line defect encircling the particle-like Saturn rings (red). (**c**) Strong anchoring condition. Another possible solution is a point defect (red dot) bearing *q* = −1.

To illustrate the derivation of this threshold condition, we consider a spherical NP of radius *R* enforcing homeotropic surface anchoring [1] (it enforces surrounding LC molecules orientation along the surface normal). In Figure 2a,b, we schematically sketch two qualitatively LC configurations, corresponding to μ≪1 and μ≫1, respectively. The key free energy density contributions describing these competing configurations are condensation and interfacial free energy densities. The case shown in Figure 2a penalises only the interfacial free energy contribution because the LC order is essentially spatially homogeneous and, consequently, it does not, on average, accommodate the homeotropic anchoring condition. Consequently, the total free energy penalty of this state is estimated by [2,40]

(12) $$F_{A}\sim 4\pi R^{2}W$$

where we use Equation (10) and W~wS. In the competing state shown in Figure 2b, the homeotropic surface anchoring condition is fulfilled. A possible scenario to establish an essentially spatially homogeneous far-nematic director field is to introduce a line defect encircling the NP, as depicted in Figure 2b. The biggest free energy penalty arises due to the essentially melted LC order within the core of the line defect. The core size of the defect is roughly given by the nematic order parameter correlation length ξ. The resulting key free energy penalty $F_{B}$ of this configuration is given by [1,40]

(13) $$F_{B}\sim 2\pi \;l\;\xi^{2}\;\left|\Delta f_{c}\right|.$$

Here, l~R stands for the radius of the ring defect, and $\left|\Delta f_{c}\right|\sim a_{0}(T_{IN}-T)S^{2}$ estimates the cost of melting the LC order within the core of the line defect. Deep in the nematic phase, it roughly holds [1] $\xi \sim \sqrt{L/(a_{0}(T_{IN}-T))}$. The balancing condition $F_{A}=F_{B}$ yields μ~1. Therefore, NP (i) distorted and (ii) non-distorted regimes, corresponding to (i) $F_{A}\gg F_{B}\;$ and (ii) $F_{A}\ll F_{B}$, refer to regimes (i) μ≫1 and (ii) μ≪1.

**Figure 3:**
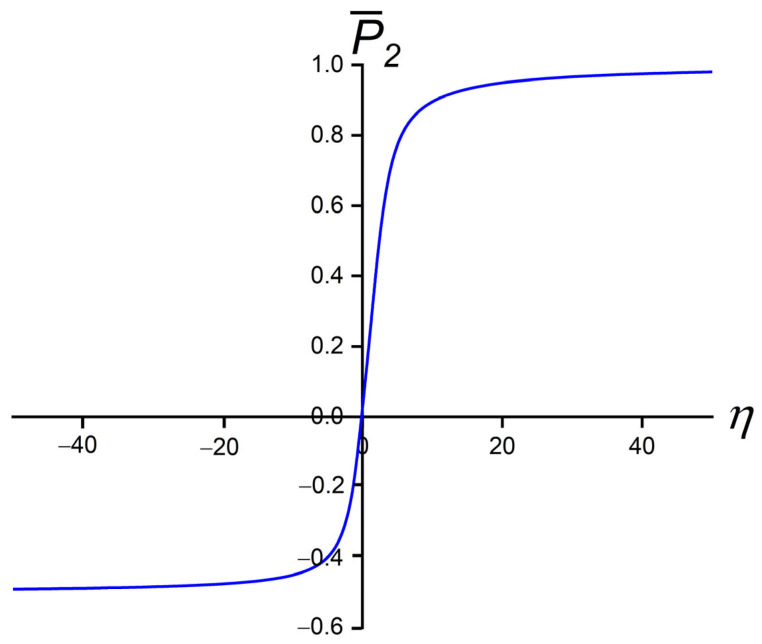
Order parameter ${\bar{P}}_{2}\;$ of strongly anisotropic NP as a function of η. In the limit η→∞, it holds ${\bar{P}}_{2}\sim 1$. In the other limit η→−∞, it holds ${\bar{P}}_{2}=-0.5$.

We next consider the impact of essentially locally homogeneous nematic order on strongly anisotropic NPs [41,42]. Let us assume that the effective interfacial contribution exhibits a field-like form given by Equation (9), where ${\hat{e}}_{i}$ describes the NP symmetry breaking direction. Therefore, the interfacial contribution integrated over the whole NP surface area $a_{NP}$ reads as follows:

(14) $$\Delta F_{i}=-w\;a_{NP}\;S\;P_{2}\left({\hat{e}}_{i}.\hat{n}\right),$$

where $P_{2}$ is the Legendre polynomial of 2nd order. The average degree of NP orientational order is determined by [42]

(15) $${\bar{P}}_{2}=\frac{\int_{0}^{\pi }P_{2}(x)e^{-\Delta F_{i}\beta }dx}{\int_{0}^{\pi }e^{-\Delta F_{i}\beta }dx}=\frac{\int_{0}^{\pi }P_{2}(x)e^{P_{2}\eta }dx}{\int_{0}^{\pi }e^{P_{2}\eta }dx}$$

where ${x=\hat{e}}_{i}.\hat{n}$, $\eta =w\;a_{NP}\;S\;\beta$, $\beta =1/(k_{b}T)$, and $k_{b}$ is the Boltzmann constant. The dependence of ${\bar{P}}_{2}$ on η is plotted in Figure 3. One sees that ${\bar{P}}_{2}(\eta >10)\sim 1$ and ${\bar{P}}_{2}\left(\eta <-10\right)\sim -0.5,$ and in the regime $\left|\eta \right|<3$, it roughly holds [42]

(16) $${\bar{P}}_{2}\sim \frac{\eta }{5}=\frac{w\;a_{NP}\;S\;\beta }{5}$$

Therefore, the degree of order of the anisotropic NP within the assumed approximation depends only on a single scaled quantity, which depends on (i) geometry, (ii) chemistry (via the anchoring strength constant), and (iii) temperature. For example, one sees that NPs exhibiting a larger surface area could be more efficiently aligned by the surrounding orientational molecular field. Furthermore, for *w* > 0 (*w* < 0), NPs display positive (negative) uniaxial order.

**Figure 4:**
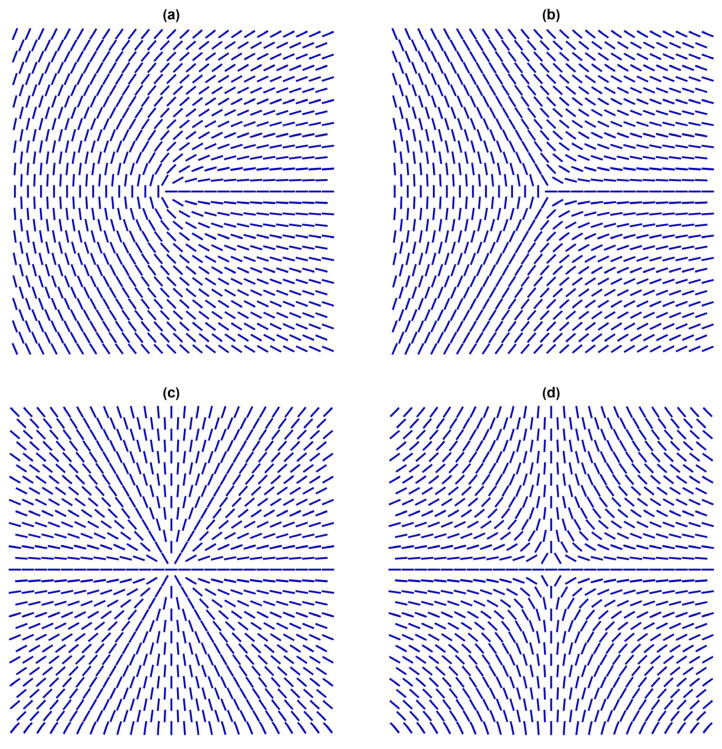
Point defects in 2D. (**a**) m=1/2; (**b**) m=−1/2; (**c**) m=1; (**d**) m=−1.

Let us assume that NPs fulfil the condition μ≫1, so they impose strong anchoring. In this case, they can act effectively as topological defects (TDs) [43,44,45]. In general, TDs correspond to localised distortions in a relevant physical field that are topologically protected. Due to their topological origin, several features dominated by TDs exhibit universal behaviours. Consequently, TDs in completely different physical systems could exhibit mathematically similar behaviour. For this reason, TDs are of interest to all branches of physics, including condensed matter, particle physics, and cosmology [31,46,47,48]. Key quantities representing TDs in the NLC order in three-dimensional (3D) space are the winding number *m* and the 3D topological charge *q*. These are discrete quantities obeying conservation laws [43,44]. The winding number $m\in \{\pm \frac{1}{2},\pm 1,\pm \frac{3}{2},\ldots \}$ measures the total circulation of $\hat{n}$ on encircling the defect centre counterclockwise along any closed curve. Note that half-integer values of *m* are possible owing to the head-to-tail axial symmetry of the NLC order. Some representative configurations of TDs possessing different values of *m* are shown in Figure 4.

Note that in 2D, only point defect can be topologically stable and, in this case, *m* is also a 2D topological charge.

**Figure 5:**
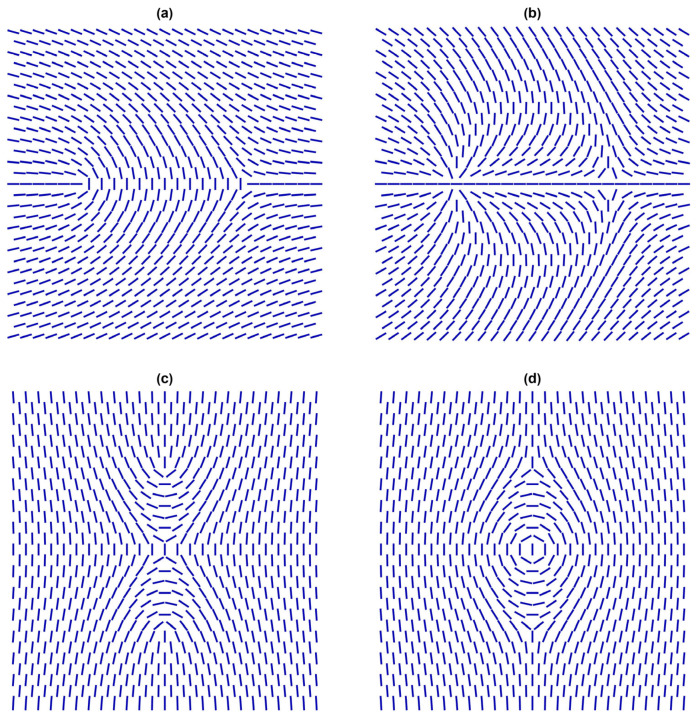
Two-dimensional defect structures where the total winding number is zero (i.e., the far-director field could be essentially spatially homogeneous). (**a**) A pair (*m*, −*m*) = (1/2, −1/2). (**b**) A pair (*m*, −*m*) = (1, −1). (**c**) A central *m* = 1 defect accompanied by two *m* = −1/2 TDs. (**d**) A central “vortex” *m* = 1 defect accompanied by two *m* = −1/2 TDs.

Figure 5 illustrates cases where the total winding number of the point TDs in the 2D plane shown equals zero. In these cases, the far-nematic director is essentially spatially homogeneous [49,50]. A defect bearing *m* > 0 and *m* < 0 is commonly referred to as *defect* and *antidefect*, respectively. Specifically, the pair {*m*, −*m*} tends to annihilate into a defect-less state. Furthermore, if one locally enforces a defect bearing $\left|m\right|>1$, it tends to decay into a sum of “elementary” defects [1,51,52], bearing $\left|m\right|\equiv \left|m_{0}\right|=\frac{1}{2},$ where the total charge is conserved. Furthermore, the 3D topological charge is in a Cartesian coordinate system defined by the equation [19]

(17) $$q=\frac{1}{4\pi }∯\hat{n}\left(\frac{\partial \hat{n}}{\partial x}\times \frac{\partial \hat{n}}{\partial x}\right)d^{2}\vec{r},$$

where the integral is carried over the surface enclosing the whole defect, and topologically stable TDs correspond to integer values of *q*. In 3D, either point or line defects could be stable. The latter are commonly presented by both topological quantities *m* and *q* [44,45]. Here, *m* yields information on the line defect structure in the plane perpendicular to the line defect local orientation. The sign of *q* defined by Equation (17) does not have physical meaning [44] because of the head-to-tail invariance of $\hat{n}.$ It is only sensible if we introduce a reference $\hat{n}$ orientation [51] and, in this case, one can treat TDs similar to in 2D. It is important to stress that line defects bearing an integer value of m are not topologically stable because they can escape along the “3rd spatial direction” [53]. A closed line defect, whose winding number switches between m=1/2 and m=−1/2 could be chargeless [50,54,55], bearing *q* = 0. The far-nematic director field of such a localised structure (which is topologically not stable) could be spatially homogeneous. Furthermore, line defects could either form closed loops or originate and terminate at an LC-limiting substrate interface [56,57,58,59]. In the case of a closed line defect, whose local structure is described by m=1/2 and m=−1/2, its far-field matches the director field of a point defect bearing $\left|q\right|=1.$

**Figure 6:**
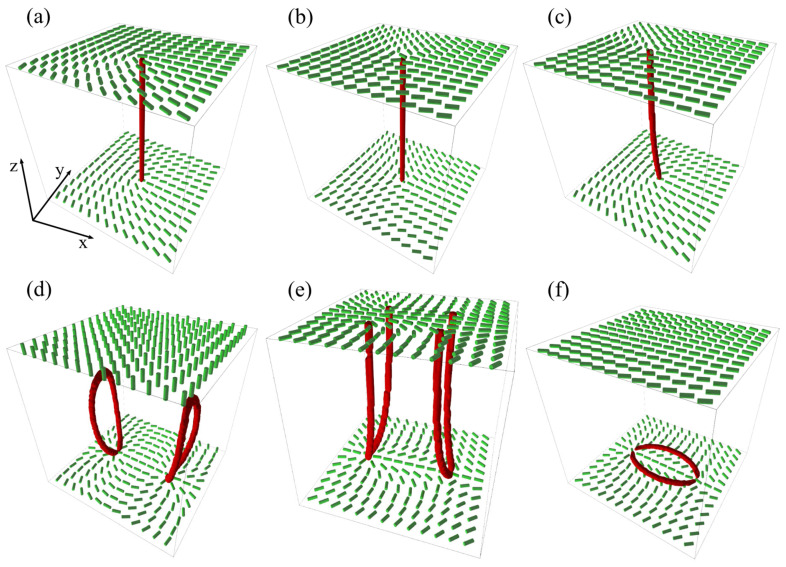
Three-dimensional NLC structures possessing line defects: (**a**) *m* = 1/2 and (**b**) *m* = −1/2 disclinations. (**c**) The so-called twist disclination possessing alternating *m* = 1/2 and *m* = −1/2 segments. In (**d**–**f**), we show three qualitatively different defect structures that can be nucleated by a limiting bottom substrate which enforces two *m* = 1 surface point defects. (**d**) Two *m* = 1/2 rings are formed, each bearing *q* = 1. In this case, a homogeneous director field along the z-direction is imposed far above the bottom plate. (**e**) Four *m* = 1/2 disclinations are formed. In this case, free boundary conditions are imposed at the top plate. (**f**) The two *m* = 1 surface point defects are joined by a pair of *m* = 1/2 disclinations.

In Figure 6, we plot some representative line defects. Suppose that the surface integral of Equation (17) is carried over the nanoparticle surface exhibiting homeotropic anchoring. In this case, the integral on the left is proportional to the Gaussian curvature; consequently, q=1−g, where *g* stands for the genus, i.e., the number of NP holes [60].

Thus, NPs of spherical topology act as TDs bearing q=1, introducing relatively strong elastic distortions to the LC medium. If such a single NP is embedded in the NLC, its charge would be compensated by an antidefect or several additional defects in such a way that the total charge of the system equals zero. For example, if a single antidefect is formed, it could exhibit either a point-like or line-like structure [56,60,61,62]. Consequently, the resulting structure consisting of the NP and the antidefect could exhibit different symmetries [31,32,41,63,64], as it is schematically depicted in Figure 2b,c.

**Figure 7:**
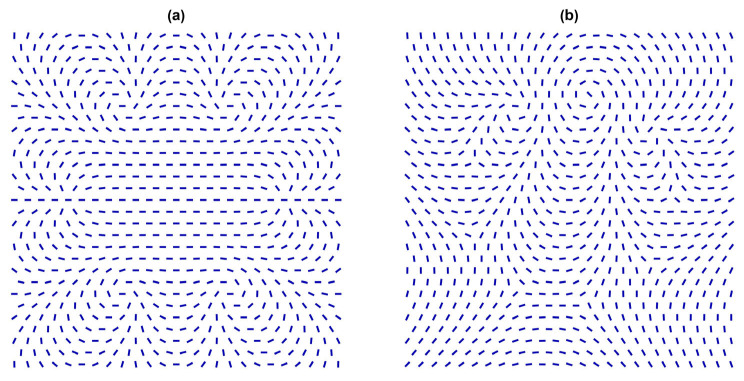
(**a**) Two-dimensional NLC structure hosting six NPs, where each of them effectively acts as an *m* = 1 point defect. The total winding number of the system equals to six. The defects mutually repel, and in the case shown, the director field in the central region is essentially spatially homogeneous. (**b**) Two-dimensional LC structure hosting three NPs, where each of them effectively acts as an *m* = 1 point defect. The total winding number of the particles is compensated for by six *m* = −1/2 point defects in the NLC body. Such a structure could be trapped in a stable or metastable configuration, which is topologically equivalent to the structure shown.

If more NPs carrying topological charges are introduced, diverse effective configurations can emerge. Some qualitatively different scenarios in 2D are illustrated in Figure 7.

In Figure 7a we illustrate a possible scenario where six TDs bearing q=1 rearrange in such a way as to form a homogeneous structure in the central region. Such cases promote phase separation [64], which one tends to avoid if homogeneous LC + NP mixtures are of interest. Note that the case shown is reminiscent [65] of the Faraday cavity effect. In the latter case, charges introduced to a 3D metal body rearrange at the outer surface in such a way that the electric field within the body equals zero to minimise the electric field energy within the body. In the case shown in Figure 7a, TDs rearrange in such a way as to minimise the elastic energy in the central region (note that $\hat{n}$ cannot be switched off in the nematic phase).

Furthermore, Figure 7b shows a possible scenario where *m* = 3 total charge is enforced by three NPs, each carrying *m* = 1. To minimise the free energy (i.e., to form a homogeneous far-nematic director field), six *m* = −1/2 are introduced to compensate for the total topological charge. Therefore, this structure is topologically equivalent to a spatially homogeneous NLC order, which cannot be achieved because the charge carried by NPs is fixed if the nematic order is not melted. The resulting structure is topologically similar to a lattice consisting of anions and cations, where the charge alternates in sign in a given direction. In this way, one achieves effectively attractive interaction. In general, if NPs strongly deform the local LC order, different structures could emerge by varying the sample’s history [64].

NLC fluctuation modes are important generators of different structural deformations [46,66,67,68,69,70,71,72]. Recent theoretical analysis by Selinger et al. [69,70] revealed that four independent modes exist in the NLC if its structure is described solely by $\hat{n}.$ These are referred to as the *bend*, *double twist*, *double splay*, and *biaxial splay*, respectively. For example, the *double twist* mode enforces the so-called double twist cylinders that can nucleate in strongly chiral LCs with different Blue Phase (BP) structures [73,74], which consist of lattices of *m* = −1/2 line defects. Note that the total NLC structure possessing such a lattice of TDs is topologically neutral (i.e., the total topological charge equals zero). Figure 5c illustrates how such neutrality is realised based on a topologically equivalent structure in a 2D system. For example, such a structure could be experimentally realised by confining a BP structure into a thin plane-parallel cell, where the confining substrates enforce isotropic tangential anchoring. The structure shown consists of one *m* = 1 and two *m* = −1/2 defects. In 3D, this structure would correspond to two m = −1/2 line defects and one nonsingular escaped *m* = 1 distortion, where the total topological winding number in any 2D plane equals zero. In strongly chiral structures, NPs could also support BP structures. For this purpose, NPs should locally relatively weakly distort $\hat{n}.$ Such NPs are attracted to the cores of line defects because they partially replace the defect core with NP volume. Specifically, the core of TDs is energetically expensive because within it, the LC order is essentially melted due to strong local elastic distortions. These effects are referred to as the Defect Core Replacement mechanism [41,75,76,77,78].

We next discuss common mechanisms that can provide history-dependent behaviour in NP–LC mixtures. In this respect, we remind that NLCs are extremely susceptible to different perturbations due to the existence of Goldstone modes in collective $\hat{n}$ excitations [1,2,3]. This sensibility is well illustrated by the Imry–Ma–Larkin [71,72] theorem, first introduced in magnetic systems. According to this theorem, even an infinitesimally weak random-field type disorder breaks a system into a domain-type pattern. The latter should exhibit short-range order. The resulting characteristic domain length $\xi_{d}^{\left(IM\right)}$ reflects the compromise between elastic and random-field free energy penalties. The former favours a spatially homogeneous nematic order. On the contrary, the random-field free energy contribution is minimised when $\hat{n}$ is aligned along a local random-field preferred direction. In the following, we present a derivation with estimates of the value of $\xi_{d}^{\left(IM\right)}$ in NLCs [79]. For the free energy, we focus on the competition between the elastic and random field contributions. Consequently, $f\sim f_{e}+f_{RF}$, where

(18) $$f_{e}\sim \frac{K}{2}{\left|\nabla \hat{n}\right|}^{2}$$

(19) $$f_{RF}=-\frac{W}{2}P_{2}\left(\hat{e}.\hat{n}\right)$$

The random-field contribution $f_{RF}$ is weighted by a positive constant W, which locally favours orientation along $\hat{e}.$ We focus on an average domain of volume $V_{d}\sim \xi_{d}^{3}$, where $\xi_{d}$ estimates the characteristic linear domain size. The corresponding average domain free energy penalty is given by

(20) $$\Delta F_{d}\sim \frac{V_{d}}{2}\left(\frac{K}{\xi_{d}^{2}}-W\bar{P_{2}\left(\hat{e}.\hat{n}\right)}\right)$$

where the overbar $\bar{\left(\ldots \right)}$ stands for the spatial average within $V_{d}.$ The central limit theorem suggests $\bar{P_{2}\left(\hat{e}.\hat{n}\right)}\sim 1/\sqrt{N_{d}}$, where $N_{d}\sim {\left(\xi_{d}/a_{RF}\right)}^{3}$ estimated number of $\hat{e}$ random reorientations within $V_{d}.$ Here, $a_{RF}$ estimates the average separation of nearby reorienting sites. The size $\xi_{d}^{\left(IM\right)}$ is obtained by balancing the elastic and random-field interactions. From the requirement $\Delta F_{d}=0$, one obtains

(21) $$\xi_{d}^{\left(IM\right)}\sim {\left(\frac{K}{Wa_{RF}^{3/2}}\right)}^{2}.$$

Furthermore, one could strongly manipulate NLC+NP configurations by varying the I–N phase transition velocity [13,80,81,82]. If it is fast enough, different system parts cannot exchange information on their local symmetry-breaking direction [31,81]. Consequently, a lack of information exchange can result in domain-type patterns, where different symmetry-breaking directions are selected in informationally decoupled LC regions. The resulting domains are characterised by a characteristic domain length $\xi_{d}$. Firstly, the formed domains are dubbed as *protodomains* [11,31]. We label their characteristic length as $\xi_{d}^{\left(p\right)}.$ After their formation, the domains grow to decrease the total area of the energetically expensive domain walls. Consequently, soon after the domains are formed, the scaling regime is entered, where the domain grows [13,80,81] as $\xi_{d}\propto t^{\gamma }$ with γ~1 in the NLCs. This growth is enabled by the mutual annihilation of defects and antidefects, which are present within the domain walls. In the following, we estimate $\xi_{d}^{\left(p\right)}$ as a function of the I–N quench rate using the Kibble–Zurek (KZ) mechanism [11,31]. It describes domain formation in systems exhibiting continuous symmetry-breaking phase transitions. In addition, it assumes a finite velocity of information propagation and considers critical slowing down if the phase transition is 2nd order or weakly 1st order. The latter case is realised in typical I–N phase transitions [1,2,3].

Originally, the KZ mechanism was derived for temperature-driven 2nd order phase transition [11,31]. The temperature variations are described by the dimensionless temperature

(22) $$r=(T-T_{c})/T_{c}$$

where $T_{c}$ stands for the phase transition temperature. In the case of I–N phase transition, the role of $T_{c}$ is played by the supercooling temperature $T^{*}.$ Let us assume linear time variation of temperature across the phase transition is characterised by the quench rate $\tau_{Q}:$

(23) $$t=-\tau_{Q}r.$$

Here, $\tau_{Q}$ describes the time needed to increase the temperature from *T* = 0 to $T_{c}{\sim T}^{*}$. Close to the phase transition, the characteristic amplitude order parameter relaxation length and relaxation time are roughly given by

(24) $$\tau \approx \frac{\tau_{0}}{{\left|r\right|}^{\eta }}\;,\;$$

(25) $$\xi \approx \frac{\xi_{0}}{{\left|r\right|}^{v}}.$$

Here, $\tau_{0}$ and $\xi_{0}$ determine characteristic responses deep in the nematic phase. Quantities η and v are critical coefficients. Typical NLCs [1,2,3,13,31,80] hold η~1 and $v\sim \frac{1}{2}$.

To estimate the size of the *protodomains,* we assume that we start from the isotropic phase, where *r* > 0 (see Equation (22)). Then, we linearly approach the phase transition. The maximal size of fluctuations exhibiting local order within the isotropic “sea” is estimated by ξ (see Equation (25)). In the time regime $\left|t\right|>\tau$, the NLC-dynamics is fast enough to adapt to changes in temperature. Therefore, the NLC order is close to its equilibrium ordering at a given temperature. Roughly, the NLC falls out of equilibrium when the time to reach the phase transition becomes comparable to the relaxation time. This time is dubbed as the Zurek time $t_{z}$ and is defined by $\left|t_{z}\right|=\tau$. From Equation (24) and Equation (23) it follows

(26) $$\left|t_{z}\right|\sim \sqrt{\tau_{0}\tau_{Q}}.$$

In the time regime $-\left|t_{z}\right|<t<\left|t_{z}\right|$ the order parameter dynamics is relatively slow. One assumes that the dynamics is frozen in this time interval. When the system exits this regime at $t=\left|t_{z}\right|$ the dynamics unfreezes. Furthermore, at $t=-\left|t_{z}\right|$ the size of the largest fluctuation generated nematic clusters is estimated by $\xi^{\left(max\right)}=\xi \left(\left|r_{z}\right|=\frac{\left|t_{z}\right|}{\tau_{Q}}\right).$ For temperatures corresponding to *t* < 0, such clusters are unfavourable. Furthermore, we assume that on crossing the temperature interval {−$\left|r_{z}\right|$,$\;\left|r_{z}\right|$} the correlation length is frozen, and it unfreezes at *t* $=\left|t_{z}\right|$. At the corresponding temperature, the clusters exhibiting orientational order become energetically favourable and tend to expand. Therefore, the initial size $\xi_{p}$ of domains, the so-called *protodomains*, are estimated by $\xi_{p}=\xi^{\left(max\right)}$. It follows

(27) $$\xi_{p}=\xi_{0}{\left(\frac{\tau_{Q}}{\tau_{0}}\right)}^{1/4}.$$

The relative magnitudes of $\xi_{d}^{\left(IM\right)}$ and $\xi_{p}$ could result in different stable or metastable NP–LC configurations. Note that in the systems of our interest, NPs could be sources of disorder, particularly in cases when (i) NPs are anisotropic, (ii) NPs interact with their surroundings in an anisotropic way (e.g., via oriented covalent bonds), or (iii) an NP and nearby NP-induced TD (or TDs) exhibit spatial anisotropy [24,25,26]. Furthermore, for fast enough quenches, when LC temporarily falls out of equilibrium, different structures are expected depending on the ratio $\frac{\xi_{d}^{\left(IM\right)}}{\xi_{p}}.$ In addition, it has to be stressed that the Imry–Ma–Larkin theorem [71,72] applies to NP–LC mixtures only for certain material conditions, which is described in detail in Ref. [79]. Consequently, a rich pallet of quantitatively or even qualitatively different effective configurations could emerge depending on the detailed geometrical and material properties [83,84,85,86,87,88,89,90] of mixtures.

## 4. Conclusions

We presented a rich diversity of configurations that could emerge from mixtures of different NPs and NLCs. The dimensionless ratio μ between the characteristic linear size of a particle and the surface interaction penetration length [19,40] well reveals the strength of mutual NLC–NP coupling. In the weak coupling regime (μ≪1), NPs immersed in the NLC host could be exploited to introduce additional material property in the effective system [4] or to stabilise lattices of disclinations in chiral LCs [75,76,77]. In the strong coupling regime (μ≫1), a richer pallet of configurations could emerge in essentially spatially homogeneous NLC–NP mixtures. In cases of strongly anisotropic NPs, which do not introduce disorder into the NLC structure, the nematic molecular field could be exploited to align the NPs along the average nematic director [39,42,91,92].

This effect could be exploited in various applications. Specifically, strongly aligned anisotropic NPs (e.g., nanotubes) could impart a strong material anisotropy into the effective system. Such a collective particle configuration could be indirectly reoriented by reorienting the hosting nematic director. Due to LC softness, the latter could be relatively easily manipulated by different external stimuli, e.g., external electric or magnetic fields, or by some other means [3].

## Data Availability

The data that support the findings of this study are available from the authors upon reasonable request.
